# Correspondence: semen parameters in men who recovered from covid-19 — a systematic review and meta-analysis

**DOI:** 10.1186/s43043-022-00115-5

**Published:** 2022-08-18

**Authors:** Diogo Edele dos Santos, Tamy Colonetti, Lidia Rosi Medeiros, Maria Laura Rodrigues Uggioni, Antonio Jose Grande, Maria Inês da Rosa

**Affiliations:** 1 Programa de Pós-Graduação em Ciências da Saúde, Laboratory of Epidemiology, Universidade do Extremo Sul Catarinense, Criciúma, SC Brazil; 2 Medical Course, Laboratory of Evidence-Based Practice, Universidade Estadual de Mato Grosso do Sul, Dourados, MS Brazil

## Abstract

A recent systematic review with meta-analysis performed by Tiwari et al. (Middle East Fertil Soc J 26:44, 2021) suggested that coronavirus disease 2019 (COVID-19) affects both semen parameters and sexual hormones. However, we have observed a few inconsistencies in their systematic review methods and their synthesis of results (meta-analysis), which would have impacted their results.

## Systematic review methods

Tiwari et al. [1] included Scopus and MEDLINE and had restricted research papers to English language. According to Cochrane Collaboration, they should have searched Embase and Cochrane Library, additionally search for gray literature and registered ongoing studies. Any missing eligible studies could have impacted the summary of the evidence.

## Synthesis of results (meta-analysis)

First, Tiwari et al. [1] included seven studies: five cross-sectional [2–6], one case control [7], and one cohort [8]. The synthesis (forest plots) are very questionable since they put in the same analysis different study designs, which may generate misleading results. Estimated intervention effects for non-randomized studies of interventions (NRSI), with different study design features, can be expected to be influenced to varying degrees by different sources of bias. Results from NRSI with different combinations of study design features should be expected to differ systematically, resulting in increased heterogeneity. As heterogeneity among NRSI is expected to be substantial due to their diversity of study designs, detected in the meta-analysis, we recommend that NRSI with different design features should be analyzed separately. Meta-analysis methods based on estimates and standard errors, and in particular the generic inverse-variance method, will be suitable for NRSI [9, 10]. They should have done a subgroup analysis for different study designs and remove the total values (diamond).

Second, most outcomes semen (semen volume, sperm concentration, total sperm number, progressive mobility, sperm motility, vitality) and sex hormones (follicle-stimulating hormone, luteinizing hormone, testosterone, prolactin and estradiol) analysis presented substantial heterogeneity in the forest plots; in one hand, metanalysis would not be recommended, because they are synthesizing different study designs and people in the same analysis. If they decided to do it, they should have done a proper sensitivity analysis and explore the sources of heterogeneity, which could be statistical, methodological, and clinical diversity among studies [11].

Third, despite their results being statistically significant, they are not clinically relevant, since the differences are very small and do not affect any sort of decision-making.

Fourth, the funnel plot presented to investigate publication bias is not recommended due to the limited number of studies included; when there are fewer studies, the power of the tests is too low to distinguish chance from real asymmetry. Thus, any assumption based on that is not valid.

In conclusion, there are major concerns about future studies being designed based in this review and not critically looking at it. The point of having a systematic review is to map the area, provide synthesis of effects, and show the best available evidence to decision-making and to plan future studies.

## Data Availability

Data sharing is not applicable to this article as no datasets were generated or analyzed during the current study.

## Abbreviations

COVID-19: Coronavirus disease 2019
NRSI: Non-randomized studies of interventions
